# Understanding Parents’ Experiences When Caring for a Child With Functional Constipation: Interpretive Description Study

**DOI:** 10.2196/24851

**Published:** 2021-01-20

**Authors:** Alison P Thompson, Shannon E MacDonald, Eytan Wine, Shannon D Scott

**Affiliations:** 1 Faculty of Nursing University of Alberta Edmonton, AB Canada; 2 Division of Gastroenterology and Nutrition, Department of Pediatrics Faculty of Medicine and Dentistry University of Alberta Edmonton, AB Canada

**Keywords:** constipation, child, parents, caregivers, qualitative research

## Abstract

**Background:**

Pediatric functional constipation (FC) is a common but serious medical condition. Despite significant effects on children, families, and the health care system, the condition is typically undertreated. Parents carry the primary responsibility for complex treatment programs; therefore, understanding their experiences and needs may offer a critical perspective toward improving clinical care.

**Objective:**

The aim of this study is to understand and give voice to parents’ experiences and information needs when caring for a child with FC. The ultimate objective is to build an evidence base suitable for creating a digital knowledge translation tool to better support parents caring for a child with FC.

**Methods:**

This qualitative design used an interpretive description methodology to generate findings aimed at improving clinical care. One-on-one, in-depth interviews were completed either in person or through web-based teleconferencing to explore parents’ perspectives. Data collection and analysis occurred concurrently.

**Results:**

Analysis of 16 interviews generated 4 major themes: *living in the shadows*; *not taken seriously*, with a subtheme of *persevering and advocating*; *missing information and misinformation*; and *self-doubt and strained relationships*. One minor theme of *affirmative influences that foster resilience and hope* was identified.

**Conclusions:**

Parents have unmet needs for support and information related to pediatric FC. To address gaps in current care provision, decision makers may consider interventions for clinicians, resources for parents, and shifting care models to better meet parents’ needs.

## Introduction

### Background

Constipation among children is common and often mistaken for a mundane nuisance rather than a serious medical condition. More than 95% of pediatric constipation cases are attributed to functional constipation (FC), which occurs without a particular medical, genetic, anatomic, or physiologic cause. Estimates are that at least 1 in 10 children worldwide is affected by pediatric FC [1,2]. FC can present with severe symptoms such as recurrent abdominal pain, painful defecation, fecal incontinence, urinary incontinence, and urinary infections. Pain, toilet avoidance, and stool withholding behaviors worsen the condition by further perpetuating fear of defecation, causing colonic dilation, and dampening neural feedback about the need to defecate. Despite being very common, pediatric FC is often underrecognized and undertreated [3]. Without effective treatment, most children develop chronic FC, with symptoms continuing through their adult years [4]. In addition, children and families experience psychological, emotional, and social consequences of FC [5-7]. For example, school attendance and peer relationships are understandably compromised by pain and incontinence. Families also report high levels of stress and decreased quality of life [5-7]. Finally, pediatric FC is a financial burden on families and health care systems [8]. Families face inflated expenses such as medications, laundry, and clothing, in addition to indirect effects such as lost income because of caregiving. Similarly, health care systems are burdened with preventable urgent care visits and high usage rates of specialist services [8,9].

Clinical practice guidelines (CPGs) describe a variety of treatment options [10-14]; however, the bulk of responsibility for implementing, monitoring, and adjusting therapies falls to parents. Certainly, clinicians can provide parents with accurate information about the condition and treatments, but improving care also requires that health care professionals move beyond their own perspective of the condition and acknowledge the unique experiences of families living with a child affected by FC. Specifically, parental experiences critically shape their information and support needs [15]. Therefore, an in-depth understanding of parents’ experiences and self-identified needs when caring for a child with FC is a necessary step to ensure that clinicians can provide *relevant* education and support. Although parental education is an important part of treatment for pediatric FC [10-12], there is a lack of research about parental perspectives of pediatric FC. A recent systematic review on the topic included only 13 studies examining parents’ experiences caring for a child with FC [16]. The primary cited limitation of the review was the small number of included studies [16]. Furthermore, there was a predominance of quantitative studies that focused on quality of life measures, which are helpful in substantiating the familial effects of childhood FC but are not optimal in understanding how health care providers can help mitigate negative experiences and outcomes [16]. Suggestions for future research include a more in-depth exploration of how to best meet parents’ information and support needs in light of the dynamic nature of the condition and its profound effects on families [16].

### Objectives

The initial aim of this study is to understand and give voice to parents’ experiences and information needs when caring for a child with FC. The ultimate objective is to build an evidence base suitable for creating a digital knowledge translation (KT) tool to better support parents caring for a child with FC.

## Methods

### Design

The study sought to answer the research question: *What are parents’ experiences and information needs when caring for a child with FC?* Because our ultimate objective was to develop knowledge that could be used to inform and improve clinical practice, we chose the interpretive description (ID) methodology [17] to foster the applicability of our results. ID methodology was developed specifically for practice-oriented sciences, to generate findings aimed at improving clinical care [17], which aligns with our pragmatic philosophical approach for this research project.

### Recruitment

Potential participants were introduced to the study through social media posts shared on child health and parenting groups (eg, Facebook, Twitter). Physical posters were also displayed in locations frequented by families (sports facilities, libraries, health care waiting rooms, etc) in a medium-sized city in Canada. The posts described the purpose of this study and the desire to speak with the parents of children with FC. In addition, we engaged in snowball sampling by asking participants whether they knew other parents who may be interested in contributing to this study. Recruitment was active from May 2019 until data collection was complete in October 2019.

### Ethical Considerations

Ethical approval from the relevant research ethics board was granted before the initiation of the study. Each potential participant received an information sheet, which provided details on the purpose of the study, identified the potential risks and benefits, and explained the voluntary nature of their participation. Participants were given an opportunity to ask questions about the research and were free to withhold consent for any reason.

All procedures were in accordance with the ethical standards of the University of Alberta Research Ethics Office (Pro00087548) and the 1964 Helsinki declaration and its later amendments or comparable ethical standards.

### Data Collection Methods

We used one-on-one, in-depth interviews to explore parents’ experiences when caring for a child with FC. The interviews were completed either in person or through web-based teleconferencing, depending on the participant’s preference and geographic location. The interviewer (AT) had experience conducting qualitative interviews and providing care as a clinician for children with FC. The interviewer did not have any pre-existing personal or professional relationships with the participants. The interviewer spoke with the participants at the beginning of the interview to discuss the reasons for conducting this research (to understand parental experiences and subsequently develop resources for parents) and to share the interviewer’s relevant clinical background—caring for families affected by pediatric FC and noting the challenges they often encountered in managing the condition. The interview style was conversational, and the participants were encouraged to discuss aspects of their experiences they deemed most important. The interviewer also used a semistructured guide (Multimedia Appendix 1) with open-ended questions. Interview questions were developed based on previous research [18-20] and clinical experience of the team. Prompts and spontaneous questions were used to facilitate participant comfort and collection of high-quality data. Interviews were recorded and transcribed verbatim by a professional transcriptionist. Data were deidentified (ie, removal of identifying data such as city names, people names, institution names) to ensure confidentiality.

### Sample

Participants were included if their child met diagnostic criteria for pediatric FC (Multimedia Appendix 2) and were willing to discuss their experiences with the interviewer. Screening was conducted by the interviewer as a preamble to the interview to ensure that participants’ stories reflected experiences of childhood FC rather than other conditions. As recruitment was most successful through web-based platforms, participants came from diverse geographical locations across North America.

On the basis of existing literature examining parental perspectives of pediatric FC and methodological recommendations, we anticipated that a sample size between 10 and 20 participants would be adequate to generate clinically significant knowledge [17,21]. The decision to end data collection was an ongoing topic of discussion within the research team and based on the processes of data analysis. Specifically, the occurrence of redundancy within the themes and rich substantiation suggested that data collection could be stopped.

### Data Analysis

We followed guidance from the applied methodology of ID [17] throughout data collection and analysis. We conducted data collection and analysis concurrently to promote data immersion as an important step toward a more thorough interpretation of experiences [17]. Interview transcripts were exported into NVivo 12 software to manage the data. Our analytic approach avoided quantification, instead of using thematic and inductive traditions [22,23]. Our analysis followed the processes of engaging with the data, organizing the data, finding patterns within the data, making sense of the patterns, and finally, developing patterns and associations into meaningful findings for applied practice [17]. The process was initiated by the first author, who also conducted the interviews, and then was verified by the author team. Reflexive journaling and field notes were used during data collection and analysis to examine potential bias, build an audit trail, and support rigor.

### Rigor

Developers of ID emphasize that the clinical expertise of researchers strengthens the design and rigor of the research [17,24]; therefore, the experiences of clinicians on our research team were seen as a benefit. One member of the research team conducted all the interviews to maintain consistency. The interview guide was reviewed by topic experts and a parent advisory group to enhance credibility and ensure that the questions could elicit meaningful information from participants. A study log was maintained during the research to document and account for methodological decisions. Data were analyzed and findings were collaboratively critiqued by the research team with the intent to develop epistemological integrity, representative credibility, analytic logic, and interpretive authority [17] to ensure high-quality research. Following ID guidance, we did not conduct member checking because of the risks of swaying interpretation and impeding the formation of meaningful clinical implications [17,25]. The study followed the Standards for Reporting Qualitative Research (SRQR) [26] (Multimedia Appendix 3).

## Results

### Overview

A total of 16 parents of children with FC provided informed consent and participated in this study. Our analysis generated 4 major themes: (1) *living in the shadows*; (2) *not taken seriously*, with a subtheme of (i) *persevering and advocating*; (3) *missing information and misinformation*; and (4) *self-doubt and strained relationships*. We identified one minor theme of *affirmative influences that foster resilience and hope*. The demographic details of the participants are presented in Table 1. All the participants in this study self-identified as caregivers with primary responsibility for managing FC. One of the parents interviewed had more than one child with FC. Participant interviews were randomly assigned a numerical code that was used as a reference marker (eg, P3) for quotes presented to support the themes in our results.

**Table 1:** Participant characteristics (N=16).

Characteristics		Participants, n (%)
**Preferred gender identity**		
	Female	16 (100)
**Number of children**		
	1	4 (25)
	2	8 (50)
	3	2 (13)
	4 or more	2 (13)
**Affected child’s age (years)**		
	3	1 (6)
	4	4 (25)
	5	4 (25)
	6	5 (31)
	7	0 (0)
	8	0 (0)
	9 or older	2 (13)
**Education level**		
	High school	1 (6)
	Postsecondary	15 (94)
**Yearly family income in Can $ (Can $1.00=US $0.78)**		
	<20,000 (15,600)	1 (6)
	20,000-40,000 (15,600–31,200)	1 (6)
	40,000-60,000 (31,200-46,800)	4 (25)
	60,000-80,000 (46,800-62,400)	2 (13)
	>80,000 (62,400)	8 (50)
**Duration of symptoms (years)**		
	<1	1 (6)
	1-2	3 (19)
	>2	12 (75)
**Number of constipation-related health care visits (total)**		
	0-5	4 (25)
	6-10	6 (38)
	More than 10	6 (38)

### Living in the Shadows

Parents in this study expressed strong feelings of isolation attributed to living with a condition that is considered taboo. Discussing bowel habits and incontinence was thought to be a difficult or inappropriate topic in social circles and within the health care context. For example, when parents themselves were open to the conversation, most had experienced or anticipated negative reactions from others. One parent related her sense of isolation, “Nobody talks about it…. So, you feel alone… And nobody wants to talk about poop*”* (P3)*.* Similarly, another parent explained:

I think, that for myself...because I don’t know a lot of other parents that are – I don’t know if people just don’t talk about it, so I don’t know how common it is.

P4

To combat feelings of isolation, parents typically searched for resources without success to meet their social support needs. Parents were surprised about the lack of discussion groups because many described how it seems there is an online forum for almost every rare disease or condition:

Something...so you’re not alone, right. Because that’s the thing and you don’t understand why your kid is having so many problems. It’s like somebody or something that explains like oh my kids have this issue, so you don’t feel like you’re the only one...Just something you can go to whether it’s like a chat group or a parent group or something.

P5

Another parent described how she would change things to improve other families’ experiences with pediatric FC:

You know, I think it’s one of those things that people could really benefit from a support group because it’s something that’s so like people don’t wanna talk about, they’re embarrassed about it.

P9

Another parent simply expressed, “I just feel like we were very much left on our own” (P14).

### Not Taken Seriously

Parents shared stories of encounters with health care professionals who did not take their concerns about constipation seriously. In some cases, parents were explicitly told that the symptoms were nothing to be concerned about, and in other cases, parents were implicitly given the impression that they were overreacting. One parent shared her care provider’s dismissive response to her child’s symptoms:

I was always told it would pass, it would pass. Probably listen to the patient a little bit better because they know their body, right, and I – me living with her, I know what’s going on with her. So, listen a little bit closer and maybe have better options than prune juice.

P13

Similarly, another parent said:

I wish I had been taken seriously right away. You know, not just like she’ll grow out of it, she’ll grow out of it. It’s normal, she’ll grow out of it. It’s like this wasn’t. I don’t know if it ever was.

P9

One shared the widespread effects of her child’s FC and the trivializing response:

I get that pediatricians are really busy with other things that are, you know, more important than constipation, but like now that he’s in school, it’s affecting his whole class. It’s affecting his teacher. It’s affecting him and his friends. Like it affects a lot of things and it affects us daily. It takes up our time as parents and his time away from his activities and the only real thing that we hear is, oh don’t worry, it’ll end soon. Like how?

14

One parent reflected on her desire for health care providers to change*:*

I guess I wish they would learn – they would take it a bit more seriously and understand how it impacts lives and how it impacts – I mean children’s lives.

P7

Parallel to instances of health care providers not taking the condition seriously, parents themselves described periods of questioning the legitimacy or validity of their own concerns. For example, one parent shared:

I think we could have maybe helped him a lot sooner if I wasn’t so scared to start the Lax-A-Day but I also didn’t want to make an appointment, take someone else’s doctor time...I hate wasting doctors time on what I consider a silly thing...I know it’s not the right way to think of it but like to my point, it had to be urgent enough.

P3

Similarly, another parent said, “You’re like, oh is that normal or not normal and you kind of doubt yourself” (P2).

#### Persevering and Advocating

As a result of symptoms and concerns not being taken seriously, parents demonstrated perseverance and became stronger advocates for their child’s health. One parent described her feelings about health care encounters:

I had talked to my doctor about it. Like our doctor and the doctor said like, oh you know, she’s still really young. She’ll grow out of it, all that kind of stuff...eventually after lots of kind of like advocating, I ended up – I was like I need another opinion on this.

P9

Similarly, another parent stated:

We found that we’ve gone to the doctor a couple of times now and they haven’t been super helpful...and then we wound up back at the doctor because we’re still – she’s still having accidents.

P16

Parents returned to health care providers repeatedly and asked for referrals to other providers because their child’s condition was worsening without adequate treatment. For example, “I’d asked many times for her to be seen by somebody else just because I need this figured out*”* (P13). Parental frustration frequently became the catalyst for advocacy. One parent expressed:

They don’t take it serious enough...it would just be nice if there was a doctor that would take you a little more serious. I know lots of kids have it and I get that, but when they get to be older and it’s a school issue, I think like we push. I think we asked – my doctor was out of town so we asked the stand in and then we asked the walk-in clinic and then we asked my doctor.

P5

### Missing Information and Misinformation

Parents caring for a child with FC frequently have unanswered questions about the condition, causes, symptoms, prognosis, and treatment. One parent said:

Maybe I wouldn’t have been so upset about it or, you know, it wouldn’t have been such an overly concern for me if I’d had a little bit more information.

P2

Similarly, another parent explained the lack of teaching provided about pediatric FC:

I’m saying like you go into the doctor and you’re like this is an issue and they don’t give you...like there’s nothing, they give you nothing. My doctor was just very much like, oh it’s super common and...like not giving you any further advice or resources.

P6

Parents frequently questioned whether there was an underlying medical cause for constipation. For example, one parent stated:

Maybe something else medically. Like maybe she’s lactose intolerant – we thought well maybe there’s some issues with milk or dairy which, of course, would not be constipation...but we were convinced it was something she was eating. Maybe it was gluten, maybe it was this, maybe it was that.

P1

Episodes of incontinence often cause parents to question the underlying reason. One parent wondered, “I don’t know if it’s medical or constipation or is it just laziness?” (P5). Similarly, another parent stated:

We had no idea whether she actually like did she have control, did she not have control. Could she feel it, could she not feel it? Was she just ignoring it? Did she need to pay more attention? Like all of these huge question marks.

P9

Questions about the treatment for pediatric FC were also common. A parent shared concerns about medication use:

You read the Lax-A-Day thing it says, “Adults only”, blah, blah, blah. So, I’m like ‘Are you sure?’ Like it feels wrong...But then, again we’re trying to cut back now on the Lax-A-Day because you can’t be on Lax-A-Day forever, can he? Like I don’t know.

P3

In addition to having questions about pediatric FC, parents shared instances of misinformation that was detrimental to their child’s care. As explored above in the theme of not being taken seriously, parents were often incorrectly told that the condition would resolve on its own. One parent shared the common false reassurances she received:

It was very much like, no, no, no, he’s fine. And it’s just constipation and he’ll grow out of it and like I feel like everybody I talked to said, he’ll grow out of it. He’ll grow out of it. He’ll grow out it. And now, two years later, he’s not growing out of it.

P14

Parents were also commonly given misinformation about dietary changes as treatment. “We were just told to increase fibre, increase water, skip the junk food, but we eat all whole foods anyways” (P4). Similarly, another parent shared, “The doctor said, it’ll get better. You know, just make sure she’s eating healthy, which she does, and it’ll get better. It’ll get better” (P16). Dietary misinformation was problematic because it was ineffective, difficult for families to manage, and delayed further treatment:

The nurse said don’t give her any dairy. And so, we were off dairy for a while and then we were off wheat for a while and it was just like a – none, none of that seemed to make much difference.

P9

Similarly, another parent reported, “Cut [cheese] out and try to increase the fruits, the vegetables, take away the bread. It was like a constant diet struggle” (P3).

Within this theme, there was one divergent case of a parent who conveyed confidence and felt that they had adequate knowledge about caregiving for a child with FC. The case had minimal health care encounters because the parent felt further support or intervention was not required. Unfortunately, the parent’s knowledge was inferred from personal experience with medical care of an unrelated population and condition, which does not align with current evidence for pediatric FC. Thus, although the participant expressed a divergent view of her experience, the data further substantiated the theme of missing information and misinformation.

### Self-Doubt and Strained Relationships

Perhaps the most resounding theme from parents’ stories was the overarching sense of frustration that developed while caring for a child with FC. One parent shared the emotional fragility that pediatric FC had created for her as a parent:

It’s pretty terrible actually. Like I should know how to deal with this. I’m a nurse. Like I was a pediatric nurse. (crying). I should know and everything that I’ve tried didn’t work and I didn’t have any guidance or any help. Like I called the doctor, well it’s you know, the pediatrician – it’s six months to get into her, so I, you know. I’m just trying things on my own. I’m googling how do you deal with this and you know, information and none of it is working and it makes me feel like – I don’t know. Like I should know how to do this, and I don’t.

P14

Self-doubt and conflict were strongly tied to the previous themes of living in the shadows, not being taken seriously, and missing information and misinformation. One parent clearly expressed the situation stating:

It was just like extremely frustrating because I felt like I wasn’t getting – I wasn’t getting enough support or information from the medical – like the health professionals we were dealing with...Like it’s so frustrating. I’m like if this is so common, why does no one have answers? – it’s just so, so frustrating.

P9

Symptoms and physiology of pediatric FC were further sources of emotional turmoil for parents:

We are very frustrated and, again, the accidents, I don’t know if it’s because of this issue or because she’s lazy or like because she’s so constipated...it’s the accidents that are driving us crazy.

P5

Another parent explained:

We’ll tell him fifteen times to go to the bathroom and he won’t and then he’ll have an accident and you feel like – you just get to your boiling point sometimes and you don’t want to yell and get angry, but sometimes you do.

P14

Finally, relationships frequently became strained as a result of pediatric FC:

It impacts a whole family dynamic, you know. Like our world, it seems like I mean this might sound dramatic, but our world has literally revolved around her bathroom habits for the last three years.

P16

Another parent expressed the strain related to behavioral interventions, “Like it’s always a fight to get her on the toilet” (P6). Another parent stated, “There’s been lots of fights. Lots of fights. Lots of I hate yous*”* (P10). Emotional burden related to pediatric FC also sparked conflict between parents and eroded parental self-efficacy:

We’re both feeling – neither one of us are confident in our parenting. So, we’re frustrated, and we can argue about it, for sure...I really felt like a failure as a mom. (pause). I don’t know and I still don’t know what to do. I don’t feel like we’re making progress and I don’t feel like I have the confidence to fix it. And then I feel like that kind of –permeates, I guess, into our whole situation. Like into everything. Like if I can’t figure out constipation, how can I figure out big things?

P14

### Affirmative Influences Foster Resilience and Hope

Despite the predominantly despondent themes that were reflected in parents’ stories, there were small but significant moments of affirmation that helped bolster parents’ confidence. This is a minor theme of our analysis because the occurrence of positive encounters and resources was unfortunately infrequent. After episodes of misinformation, accurate and understandable explanations of the condition and symptoms were critically important for parents:

They explained the encopresis is like the fact that like you know, when she did get constipated, the accidents would just be like the new poop coming around the old stuff that’s not coming out...it’s just like your muscles are just weak because like they’ve been holding it for so long. Yeah, and I was just like – at first, it just kinda blew my mind and I’m like, why the hell has no one told me about this?

P9

Validation came from a variety of sources and was always highlighted as an important event within the caregiving experience. For example, one parent found support through the school system:

And it was really just brushed off and it’s still being brushed off until like finally – now that he’s taking up so much time from his teacher, the principal has become involved and she has been our only real advocate and our only – like the principal of the school. Like she’s not a health care provider. You know, like she’s the only person that has really like tried to help at all.

P14

Parents identified encounters that met their support and informational needs as turning points that rekindled hope and buoyed their confidence. Unfortunately, affirmative influences were meaningful but scarce in parents’ experiences. Specifically, many parents did not relate any positive encounters or support at all throughout their caregiving journey. One parent explained:

I told them this has been an ongoing issue. This isn’t getting any better. This isn’t an issue we’ve had for six months. This is an issue we’ve had for over three years now.

P16

## Discussion

### Principal Findings

Findings from our exploration of parents’ experiences with pediatric FC parallel and expand upon results from previous research in the field. In a 2003 study, researchers examined parents’ health care encounters related to childhood constipation and found similar themes of “dismissed and fobbed off, asserting the need for action, and validation and acknowledgment” [21]. The continuity of these findings with ours suggests that parents’ perceptions of encounters with health care providers related to pediatric FC have not improved significantly over the last 17 years. Despite the widespread prevalence of the condition [1,2] and advances in understanding childhood FC [3,4], parents’ concerns continue to be minimized and clinicians’ treatment discussions lag behind or are incongruent with symptom severity. In other words, when health care providers acknowledge that pediatric FC requires treatment (which in itself may occur belatedly, if at all), the level of intervention is often inadequate for the advanced nature of symptoms described by parents.

Similar to exploring patient and family experiences, measuring quality of life is considered an important way to understand the effects of a health condition or treatment on “patients’ lives, rather than just on their bodies” [27]. Numerous studies have highlighted the diminished quality of life of parents and families living with pediatric FC [28-32]. For example, 3 studies found that increased family conflict, impaired family functioning, and increased parental worry or stress were related to the presence of fecal incontinence [29,30,32]. Furthermore, Wang et al [31] found that the caregivers of children with FC gave lower ratings of their daily activities and family relationships, in addition to reporting lower physical, emotional, social, cognitive, and communication scores compared with those of the caregivers and families with healthy children. Although quality of life data provide a broad assessment of the effects of a health condition and are a central contribution to the field, qualitative methods are helpful in adding important context by exploring why and how families are affected. In this study, parental perspectives provide insights into the significant physical, emotional, and psychological burden on caregivers. Parents’ feelings of isolation and frustration were related to incontinence and further compounded by nonsupportive interactions and misinformation. Parents’ experiences of being told erroneously that pediatric FC would resolve, feeling blamed for the condition or lack of treatment success, and struggling to talk about the condition may help explain the widespread and profound impairments in quality of life for families affected by pediatric FC [28-32].

A 2019 study examining the prevalence of defecation disorders in children concluded that childhood constipation is likely underestimated by parents who may not consider symptoms sufficient to be labeled a medical condition [2]. The findings seem to be in contrast to our data, which found that parents were more frequently dismissed by health care providers rather than they being dismissive of the child’s symptoms. One potential explanation for this difference could be the relative disease severity of the parents surveyed in the 2 studies. Specifically, the cross-sectional study included a random selection of parents from the general population and was, therefore, more likely to include parents with early or mild manifestations compared with parents included in this study whose children all met full diagnostic criteria for pediatric FC. The findings from this study offer a relevant counterpoint, meaning that although parents and families may underestimate early symptoms, once the magnitude of the condition becomes evident, health care providers may be more of a barrier to recognition and diagnosis than parents.

### Clinical Implications

Our exploration of parents’ experiences of caring for a child with FC provides important insights toward improving clinical care for this difficult condition. CPGs, which are intended to support clinicians and optimize care, identify family education about pediatric FC as a key component of treatment [10-12]. Unfortunately, our results suggest that this step is commonly missing in health care encounters and that some providers even contribute to misinformation. As our data were focused on parental perspectives, we cannot report the reasons for CPG deviations. Given the time-consuming nature of consultations to provide emotional support and education, it is possible that care providers may be tempted to defer, rush through, or simply struggle to fit these practices into already-busy schedules. On the basis of parents’ reluctance to initiate discussions about bowel concerns, it may be prudent for professionals to recognize that effects may be more severe and have persisted for a significant duration by the time these issues are brought to their attention. In contrast to the temptation to offer hasty reassurance, clinicians may need to reframe their thinking toward acknowledgment, education, and active treatment. For example, explaining that the condition is common can be a method of validating parents’ concerns and mitigating parental feelings of guilt but should not be conflated by suggesting that the symptoms are normal or do not require treatment. Improving the quality of health care encounters may require education or interventions to improve the responses and treatment knowledge of health care providers. Similar to findings from a previous study about medication adherence [33], parents commonly expressed a lack of information about medication use; therefore, discussions about dosing, duration of use, side effects, and safety are likely to be well received by parents. Finally, clinicians should be attuned to inquiring about parental experiences of isolation and lack of social support during assessment and include these factors as part of treatment plans [10-12]. In addition to the existing system constraints that disincentivize lengthy consultations, it is unlikely that specialty care providers or primary care clinicians alone can adequately meet complex parental needs. Consideration of alternative care models, such as integration of nursing and allied health members, may be helpful to more accurately and consistently meet parents’ support needs when caring for a child with FC [34-36].

### Future Steps

The results of this study are an important foundation for creating resources that directly address parents’ experiences and self-identified needs when caring for a child with FC. Developing support such as digital KT tools that target parents’ information needs may improve families’ experiences of living with pediatric FC. For example, parents seek answers to concrete questions about medication dosing, titration, side effects, safety, and long-term use. Sharing information with parents about digestive physiology, including how constipation can contribute to fecal incontinence, may be helpful in empowering parents’ caregiving when faced with the uncertainty and frustration that arise from a child’s stool accidents. In addition, the emotional toll of pediatric FC on families was often underacknowledged, wherein parents’ caregiving abilities were hindered because of self-doubt and guilt. Creating resources that validate parental concerns and experiences can be an important contribution to meeting the support needs of parents caring for a child with FC. Finally, in light of our findings related to health care providers, future research exploring health care professionals’ knowledge of pediatric FC and their experiences working with affected families can clarify the challenges and barriers to improving care provision for this condition.

### Limitations

Although the recruitment was open to all parents, we only received interest from mothers. The interviewer asked whether any other caregivers from each family would be interested in sharing their perspective; however, we did not successfully recruit any further participants. Therefore, our results may not reflect the experiences of fathers and nonprimary caregivers. Parents who shared their story for this study were typically from higher education and income levels; therefore, experiences of parents with lower levels of education or income may not be adequately captured in our findings. In addition, the sample may reflect bias because of the self-selection nature of the recruitment process.

### Conclusions

Understanding parents’ experiences when caring for a child with FC is an important and often overlooked step toward improving care for this difficult condition. Our findings indicate that parents have significant unmet needs for support and information related to pediatric FC. To address gaps in current care provision, decision makers may consider interventions for clinicians, resources for parents, and shifting care models to better meet parents’ needs.

## Appendix

Multimedia Appendix 1 Draft interview guide.

Multimedia Appendix 2 ROME IV diagnostic criteria for pediatric functional constipation.

Multimedia Appendix 3 Standards for Reporting Qualitative Research (SRQR) checklist.

## Abbreviations

CPG: clinical practice guidelines
FC: functional constipation
ID: interpretive description
KT: knowledge translation
