# Supplementary material for: Understanding Parents’ Experiences When Caring for a Child With Functional Constipation: Interpretive Description Study
Source: JMIR Pediatr Parent. 2021 Jan 20;4(1):e24851. doi: 10.2196/24851 (PMC7857943; doi:10.2196/24851)
Supplement: Multimedia Appendix 1 [file pediatrics_v4i1e24851_app1.pdf]

DRAFT INTERVIEW GUIDE*

Project Title - Collaborating with Parents to Understand and Address Information Needs when Caring for a Child with Functional Constipation

1. Tell me about your experiences of having a child with constipation?
2. What did you understand about constipation when your child was first diagnosed?
    - What information were you provided with from health care providers when your child was first diagnosed? What, if anything, was helpful about this information?
    - When did they give you this information?
    - In what format did the health care providers give you this information? Written, verbal, demonstration, combination?
3. How would you have liked to receive this information?
4. How did this information influence your experience with your child's illness?
5. If your friend's child had constipation and she asked you for information, what would you teach her about it?
    - When would you give her this information?
6. Today, do you feel you have enough information about your child's constipation?
    - If no, what would you like more information on?
7. How have your learning needs changed over time (comparison between time of diagnosis and now?)
    - Better access to information?
    - Relationship with health care personnel?
8. How has your confidence to manage your child's illness changed over time? What has influenced your confidence level over time?
9. What was has been the hardest part of having a child with constipation? How has that changed over time?
10. What would you like health providers to know about your experiences of living with a child who has constipation?
11. What is important to you in terms of your child's health outcomes?
12. How do you "define" when your child has a good day? E.g. Number of stools per day? No complaints of pain? No soiling? Child attending school or activities? Parents being able to go to work?

Thank you for your thoughtful feedback to my questions. Do you have any questions or concerns?
