# Supplementary material for: Understanding Parents’ Experiences When Caring for a Child With Functional Constipation: Interpretive Description Study
Source: JMIR Pediatr Parent. 2021 Jan 20;4(1):e24851. doi: 10.2196/24851 (PMC7857943; doi:10.2196/24851)
Supplement: Multimedia Appendix 2 [file pediatrics_v4i1e24851_app2.pdf]

ROME IV Diagnostic Criteria for Functional Constipation in Infants & Toddlers

Must include 1 month of at least 2 or more of the following in infants up to 4 years of age:
2 or fewer defecations per week
History of excessive stool retention
History of painful or hard bowel movements
History of large diameter stools
Presence of a large fecal mass in the rectum
In toilet-trained children, the following addition criteria may be used:
At least 1 episode/week of incontinence after the acquisition of toilet skills
History of large diameter stools that may obstruct the toilet

Benninga MA, Nurko S, Faure C, Hyman PE, St. James Roberts I, Schechter NL. Childhood Functional Gastrointestinal Disorders: Neonate/Toddler. Gastroenterology. 2016;150:1443-1455.e2.

ROME IV Diagnostic Criteria for Functional Constipation in Children

Must include 2 or more of the following occurring at least once per week for a minimum of 1 month with insufficient criteria for a diagnosis of irritable bowel syndrome:
2 or fewer defecations in the toilet per week in a child of a developmental age of at least 4 years
At least 1 episode of fecal incontinence per week
History of retentive posturing or excessive volitional stool retention
History of painful or hard bowel movements
Presence of a large fecal mass in the rectum
History of large diameter stools that can obstruct the toilet
After appropriate evaluation, the symptoms cannot be fully explained by another medical condition

Hyams JS, Di Lorenzo C, Saps M, Shulman RJ, Staiano A, van Tilburg M. Childhood Functional Gastrointestinal Disorders: Child/Adolescent. Gastroenterology. 2016;150:1456-1468.e2.
